# FNA-based lipidomics reveals coordinated lipid metabolic remodeling in pancreatic cancer

**DOI:** 10.3389/fmolb.2026.1823383

**Published:** 2026-05-29

**Authors:** Shenhao Liu, Lifeng He, Gelin Jiang, Songling Lei, Bin Xu, Jian Xu, Songmei Lou

**Affiliations:** 1 School of Medical Technology and Information Engineering, Zhejiang Chinese Medical University, Hangzhou, China; 2 Zhejiang University School of Medicine Sir Run Run Shaw Hospital, Hangzhou, China

**Keywords:** EUS-FNA, LC-MS/MS, lipidomics, metabolic reprogramming, micro-biopsy, PDAC

## Abstract

**Background:**

Pancreatic ductal adenocarcinoma (PDAC) is a highly aggressive malignancy with poor prognosis. Endoscopic ultrasound-guided fine-needle aspiration (EUS-FNA) is routinely used for preoperative tissue confirmation; however, its potential for comprehensive lipidomic profiling in a preoperative diagnostic setting remains insufficiently explored. Given the critical role of lipid metabolic reprogramming in PDAC progression, we investigated whether lipidomic alterations could be reliably captured in EUS-FNA–derived specimens.

**Methods:**

Paired tumor and adjacent non-tumor tissues obtained via EUS-FNA from 13 PDAC patients were subjected to widely targeted (pseudo-targeted) liquid chromatography–tandem mass spectrometry (LC–MS/MS)–based lipidomic analysis. Differential lipid species were identified through multivariate and univariate analyses. A composite lipid score was constructed based on principal component loadings. Serum samples from an independent cohort of 30 PDAC patients were included for exploratory projection analysis.

**Results:**

A total of 1822 lipid species across 47 lipid classes were detected in EUS-FNA–derived specimens. Tumor tissues displayed coordinated lipid alterations, including accumulation of storage lipids and structural remodeling of fatty acyl chains characterized by elongation and increased unsaturation. These alterations were readily detectable in EUS-FNA–derived specimens. These patterns showed a tendency to distinguish tumor from adjacent non-tumor samples within the FNA cohort. Exploratory projection suggested directionally consistent lipid changes in serum samples.

**Conclusion:**

Lipid metabolic remodeling in PDAC can be reliably detected in preoperative EUS-FNA–derived specimens. These findings support the feasibility of lipidomic profiling in minimally invasive diagnostic samples and highlight the translational potential of EUS-FNA–based metabolic assessment.

## 1 Introduction

Pancreatic ductal adenocarcinoma (PDAC) is a highly aggressive malignancy associated with an extremely poor prognosis and limited treatment options (Siegel et al., 2024). PDAC is currently the third leading cause of cancer-related death, with a 5-year survival rate of approximately 13% (Siegel et al., 2025). Pathological confirmation is required for the diagnosis of suspected pancreatic lesions, particularly when therapeutic decisions depend on tissue characterization. Endoscopic ultrasound-guided tissue acquisition, including fine-needle aspiration (EUS-FNA) and fine-needle biopsy (EUS-FNB), provides a minimally invasive approach for obtaining cytological and histological specimens under real-time imaging guidance (Iwashita et al., 2024; Tempero et al., 2021). Histopathological examination of biopsy material remains essential for definitive diagnosis. In this context, EUS-FNA remains a routinely used approach in many clinical settings due to its accessibility and suitability for cytopathological assessment. It allows repeated sampling with relatively low patient burden and provides a practical platform for both diagnosis and downstream molecular analyses (Hewitt et al., 2012; Manthopoulou et al., 2023; Antonova et al., 2024).

Based on these advantages, systematic profiling of lipid composition in EUS-FNA specimens could provide valuable reference data for understanding tumor biology and exploring potential diagnostic applications. Beyond cytopathological diagnosis, EUS-FNA specimens are increasingly utilized for molecular analyses, including genomic profiling (Maher et al., 2025; Ashida and Kitano, 2022). With the advancement of high-sensitivity mass spectrometry technologies, comprehensive omics characterization from limited biopsy material has become technically feasible. Particularly under conditions of limited tissue input, the diagnostic potential of lipidomics in EUS-FNA-derived specimens has not yet been fully evaluated.

Metabolic reprogramming, particularly alterations in lipid metabolism, has emerged as a characteristic feature of PDAC (Halbrook and Lyssiotis, 2017; Kamphorst et al., 2015). Previous lipidomic studies have reported alterations in cholesterol metabolism, enrichment of long-chain unsaturated fatty acids, and global remodeling of lipid profiles in pancreatic cancer tissues (Naudin et al., 2023; Wolrab et al., 2022; Horejsi et al., 2023; Perazzoli et al., 2023). In addition to tissue-based analyses, recent studies have also explored lipidomic profiling in peripheral blood and plasma-derived extracellular vesicles, highlighting the feasibility of minimally invasive lipid-based biomarker discovery (Biancur et al., 2017; Wang et al., 2021; Cho et al., 2026). While informative, these materials represent either postoperative tumor tissue or systemic metabolic alterations and may not capture the localized metabolic features present in preoperative biopsy samples and therefore cannot directly inform preoperative clinical decision-making.

Recent advances in high-resolution LC–MS/MS technologies have enabled comprehensive lipidomic profiling from limited tissue samples (Wenk, 2005). Lipidomics offers high analytical sensitivity and the ability to capture global structural features of lipid species, enabling systematic characterization of metabolic alterations (Shevchenko and Simons, 2010). These technical developments make lipidomic analysis theoretically compatible with micro-scale biopsy specimens. However, the feasibility and reliability of lipidomic profiling in EUS-FNA-derived samples have not yet been systematically evaluated.

However, existing lipidomic studies based on resected tissues or circulating samples do not adequately capture tumor-associated metabolic features at the time of initial diagnosis, particularly in preoperative biopsy settings encountered during clinical decision-making. Therefore, in this study, we sought to determine whether lipidomic remodeling can be reliably detected in preoperative EUS-FNA-derived specimens from patients with PDAC and whether such lipidomic signatures may provide potential complementary information beyond conventional cytopathological assessment. Using pseudo-targeted LC–MS/MS, we systematically profiled lipidomic alterations in paired tumor and adjacent non-tumor biopsy samples, examined structural remodeling patterns such as fatty acyl chain length and unsaturation, and evaluated the potential diagnostic relevance of lipid signatures through multivariate modeling and ROC analysis. By integrating high-sensitivity mass spectrometry with micro-biopsy material, this work suggests that EUS-FNA–derived micro-biopsy samples can preserve and capture tumor-associated lipidomic features, supporting their applicability for preoperative metabolic assessment.

## 2 Materials and methods

### 2.1 Patient samples

Paired tumor and adjacent non-tumor pancreatic tissues were obtained via endoscopic ultrasound-guided fine-needle aspiration (EUS-FNA) from patients diagnosed with pancreatic ductal adenocarcinoma (PDAC) at Sir Run Run Shaw Hospital between January 2024 and January 2025. Only samples meeting the predefined quality and quantity requirements for LC–MS-based lipidomic analysis were included, resulting in a final cohort of 13 paired samples. Histopathological confirmation was established based on examination of FNA-derived specimens. Written informed consent was obtained from all participants, and the study protocol was approved by the Institutional Ethics Committee (Approval No. 2024-0593).

Serum samples were collected from an independent cohort of 30 patients with confirmed PDAC during the same period. Lipidomic profiling was performed using the same LC–MS platform and analytical workflow as applied to the FNA specimens. For exploratory analysis, serum lipid levels corresponding to the FNA-derived signature were standardized using the mean and standard deviation derived from the FNA cohort and subsequently evaluated within the principal component framework defined by the FNA data.

### 2.2 Lipid extraction and LC-MS/MS analysis

Approximately 20 mg of each tissue sample was weighed and homogenized using a ball mill (30 Hz, 20 s). Lipids were extracted by adding 1 mL of methyl tert-butyl ether (MTBE): methanol (3:1, v/v) containing internal standards, followed by vortexing for 15 min. Subsequently, 200 µL of ultrapure water was added, vortexed for 1 min, and centrifuged at 12,000 rpm for 10 min at 4 °C. The upper organic phase was collected and evaporated to dryness under vacuum. Dried extracts were reconstituted in 200 µL of acetonitrile:isopropanol (1:1, v/v) for subsequent analysis.

Lipid profiling was performed using a QTRAP® 6500+ LC-MS/MS system (SCIEX, United States) coupled with an ExionLC™ AD UPLC system. Separation was achieved on a Thermo Accucore™ C30 column (2.1 × 100 mm, 2.6 µm) maintained at 45 °C. The mobile phase consisted of solvent A (acetonitrile:water, 60:40, v/v, with 0.1% formic acid and 10 mM ammonium formate) and solvent B (acetonitrile:isopropanol, 10:90, v/v, with 0.1% formic acid and 10 mM ammonium formate). A gradient elution program was applied at a flow rate of 0.35 mL/min over 20 min. The injection volume was 2 µL.

Data acquisition was performed in both positive and negative ion modes using multiple reaction monitoring (MRM). The electrospray ionization source was operated at 500 °C with an ion spray voltage of +5500 V/-4500 V. Curtain gas, ion source gas 1, and ion source gas 2 were set at 35 psi, 45 psi, and 55 psi, respectively.

### 2.3 Widely targeted lipidomics analysis

Lipidomic analysis was performed on a UPLC–MS/MS platform using a widely targeted (pseudo-targeted) lipidomics strategy based on a self-built database (Metware, Wuhan, China), as previously described (Du et al., 2024). Lipid identification was achieved by matching retention time (RT) and precursor–product ion pairs.

For quantitative analysis, multiple reaction monitoring (MRM) mode was employed to detect predefined lipid features. The MRM transitions were derived from database-integrated ion pair information rather than being individually optimized for each lipid species. Representative MRM transitions (Q1/Q3) for selected lipid species are provided in Supplementary Table S3, covering major lipid classes and key differential lipids identified in this study.

Following data acquisition, chromatographic peak areas were integrated and normalized using internal standards to obtain relative quantitative results.

### 2.4 Data processing and quality control

Raw data files were processed using Analyst 1.6.3 software (SCIEX). Total ion chromatograms (TIC) were assessed for signal stability, and extracted ion chromatograms (XIC) were used for integration. Quality control (QC) samples, prepared by pooling aliquots from all specimens, were analyzed periodically throughout the sequence. QC performance was evaluated by calculating Pearson correlation coefficients and coefficient of variation (CV) values across replicates.

Lipid levels were normalized based on internal standard signals and expressed as semi-quantitative values (nmol/g tissue). Data were log2-transformed and subjected to unit variance scaling prior to statistical analysis.

The reported lipid levels (nmol/g tissue) represent semi-quantitative estimates based on internal standard normalization rather than absolute concentrations.

### 2.5 Statistical analysis

Statistical analyses were performed using R (version 3.5.1) and the MetaboAnalystR package (version 1.0.1). Principal component analysis (PCA) and orthogonal partial least squares discriminant analysis (OPLS-DA) were conducted to evaluate overall lipidomic variation between tumor and adjacent non-tumor samples. Model robustness of OPLS-DA was assessed using permutation testing.

Differential lipid species were identified based on variable importance in projection (VIP >1) and Student’s t-test. P-values were adjusted for multiple comparisons using the Benjamini–Hochberg method, and adjusted P < 0.05 was considered statistically significant.

Kyoto Encyclopedia of Genes and Genomes (KEGG) pathway enrichment analysis was performed to explore pathways associated with differential lipid species.

### 2.6 Receiver operating characteristic (ROC) analysis

Receiver operating characteristic (ROC) curve analysis was performed using the pROC package in R (version 3.5.1) to evaluate the discriminative ability of differential lipid metabolites between tumor and adjacent non-tumor samples. For each lipid species, the area under the curve (AUC) was calculated with corresponding 95% confidence intervals. Lipid species with AUC values greater than 0.70 were considered to exhibit moderate discriminative potential.

## 3 Results

### 3.1 Cohort overview

**FIGURE 1:**
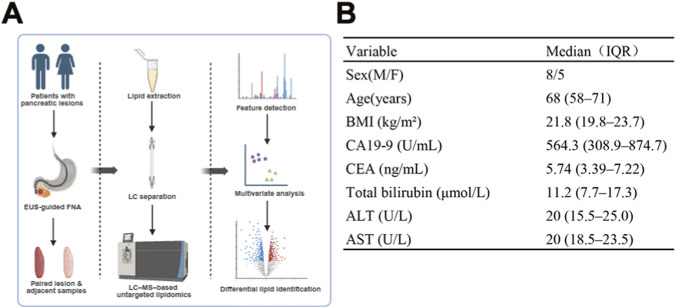
Study design and clinical characteristics of patients undergoing EUS-FNA–based lipidomic profiling. **(A)** Schematic overview of the study workflow. Paired tumor and adjacent non-tumor tissues were obtained via endoscopic ultrasound-guided fine-needle aspiration (EUS-FNA) from patients with pancreatic lesions. Lipids were extracted and analyzed using LC–MS–based lipidomic profiling, followed by feature detection, multivariate analysis, and differential lipid identification. **(B)** Baseline clinical characteristics of the study cohort, presented as median (interquartile range).

A total of 43 patients with PDAC were included in this study. Among them, 13 patients underwent EUS-guided fine-needle aspiration (EUS-FNA), from whom paired tumor and adjacent non-tumor specimens were obtained for lipidomic profiling. In addition, serum samples were collected from 30 independent PDAC patients for exploratory analysis. The study design and baseline clinical characteristics are summarized in Figure 1. We first assessed analytical performance and data quality prior to downstream lipidomic analyses.

### 3.2 Data quality assessment

**FIGURE 2:**
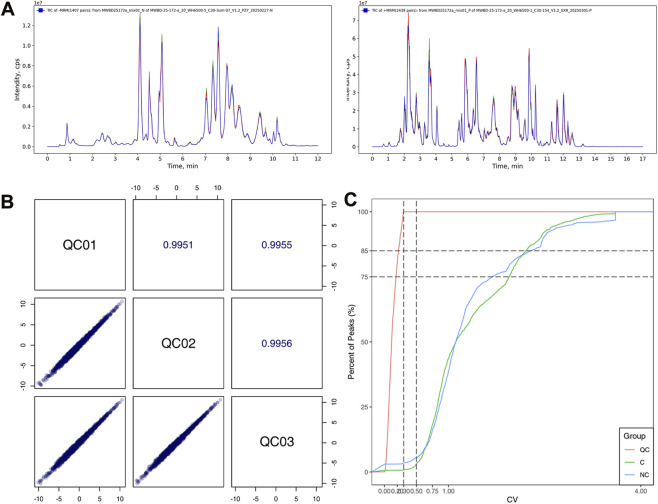
Data quality assessment of lipidomic profiling. **(A)** Total ion chromatograms (TIC) of representative EUS-FNA samples acquired in negative ion mode (upper) and positive ion mode (lower), demonstrating consistent chromatographic performance. **(B)** Correlation matrix of quality control (QC) samples, showing high reproducibility across replicates (Pearson correlation coefficients >0.99). **(C)** Cumulative distribution of coefficient of variation (CV) across all detected lipid species.

The total ion chromatograms (TIC) of all samples demonstrated consistent signal intensity and retention time across the batch acquisition (Figure 2A). Quality control (QC) samples showed high reproducibility, with Pearson correlation coefficients exceeding 0.99 (Figure 2B). The coefficient of variation (CV) distribution indicated excellent analytical stability in QC samples, with the majority of features exhibiting CV values below 0.3. In contrast, increased variability was observed in clinical samples, which is expected given the inherent biological heterogeneity of PDAC and the limited input material associated with EUS-FNA-derived specimens (Figure 2C). No strict CV-based filtering threshold was applied in this study. Given the characteristics of EUS-FNA-derived samples, applying stringent CV filtering may lead to the exclusion of potentially informative lipid features.

### 3.3 Global lipidomic profiling

**FIGURE 3:**
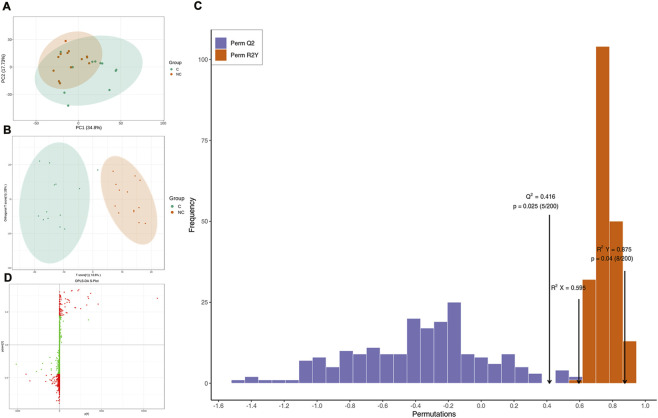
Multivariate analysis of lipidomic profiles in PDAC and adjacent non-tumor tissues. **(A)** Principal component analysis (PCA) score plot showing substantial overlap between tumor (C) and non-tumor (NC) samples, with a limited trend of differentiation. **(B)** Orthogonal partial least squares discriminant analysis (OPLS-DA) score plot showing a tendency to distinguish between groups. **(C)** OPLS-DA permutation test indicating model stability (R^2^Y = 0.875, Q^2^ = 0.416). **(D)** S-plot of OPLS-DA illustrating the contribution of individual lipid variables to group differentiation.

A total of 1822 lipid species across 47 lipid classes were detected and profiled in all paired tumor and adjacent non-tumor samples. Detailed lipid information is provided in Supplementary Table S1 and Supplementary Table S2. Principal component analysis (PCA) revealed substantial overlap between tumor and adjacent non-tumor samples, with only a limited trend of differentiation observed (Figure 3A). Orthogonal partial least squares discriminant analysis (OPLS-DA) showed a tendency to distinguish between groups (Figure 3B). The permutation test confirmed model validity, with R^2^Y = 0.875 and Q^2^ = 0.416 (Figure 3C), although the moderate Q^2^ value suggests limited predictive capacity. Together, these analyses suggest that lipidomic profiles derived from EUS-FNA samples retain biologically relevant signals, although their discriminative performance should be interpreted with caution. An S-plot was further generated to visualize the contribution of individual lipid variables to group differentiation (Figure 3D).

### 3.4 Lipid subclass composition and distribution

**FIGURE 4:**
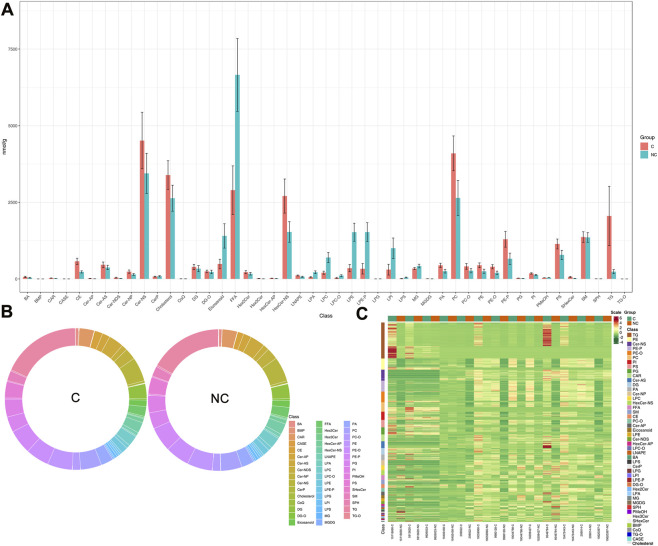
Lipid class distribution and composition in PDAC and adjacent non-tumor tissues. **(A)** Bar plot of the total content of each lipid class, demonstrating significant differences in triglycerides (TG), phosphatidylcholine (PC), cholesterol ester (CE), and free fatty acids (FFA) between groups. **(B)** Ring charts showing relative proportions of lipid classes in tumor (C) and non-tumor (NC) samples. **(C)** Heatmap illustrating differential abundance of individual lipid species across all samples.

Quantitative comparison of lipid classes revealed marked differences between tumor and adjacent non-tumor tissues (Figure 4A). Triglycerides (TG), phosphatidylcholine (PC), and cholesterol esters (CE) exhibited higher levels in tumor samples, while free fatty acids (FFA) were relatively decreased, suggesting altered lipid storage and utilization. Ring charts further demonstrated distinct lipid class compositions, with tumor tissues showing an increased relative proportion of TG and CE compared to non-tumor controls (Figure 4B). Hierarchical clustering heatmap visualized the differential abundance of individual lipid species, supporting the divergence in lipidomic profiles across all paired samples (Figure 4C). Notably, lipid alterations were not randomly distributed across classes but predominantly enriched in storage lipids and membrane phospholipids, suggesting a pattern consistent with coordinated remodeling of lipid metabolism rather than isolated perturbations, which can be captured in micro-biopsy samples obtained via EUS-FNA.

### 3.5 Fatty acid chain length and unsaturation patterns

**FIGURE 5:**
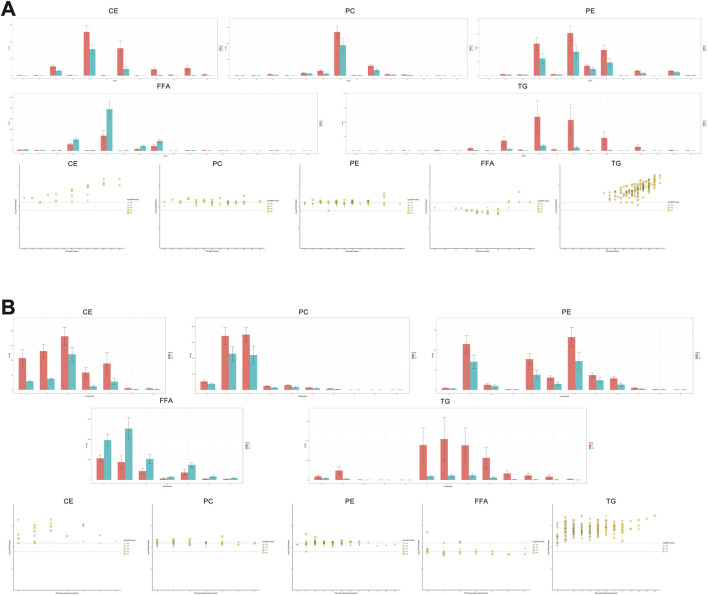
Chain length and unsaturation characteristics of major lipid classes. **(A)** Carbon chain length distribution of cholesteryl esters (CE), phosphatidylcholine (PC), phosphatidylethanolamine (PE), free fatty acids (FFA), and triglycerides (TG) between tumor (C) and non-tumor (NC) samples. **(B)** Double bond content distribution of cholesteryl esters (CE), phosphatidylcholine (PC), phosphatidylethanolamine (PE), free fatty acids (FFA), and triglycerides (TG), illustrating differences in unsaturation patterns across groups.

The carbon chain length distribution analysis revealed that tumor tissues exhibited a higher proportion of longer-chain triglycerides (TG) and phosphatidylethanolamine (PE) species compared to adjacent non-tumor tissues (Figure 5A). In contrast, free fatty acids (FFA) in tumor samples showed a relative enrichment of shorter-chain components. Analysis of double bond content demonstrated increased unsaturation in TG and PE in tumor tissues, whereas phosphatidylcholine (PC) and cholesteryl esters (CE) displayed comparable unsaturation patterns between groups (Figure 5B). These findings indicate that both chain length elongation and altered unsaturation contribute to the distinct lipidomic remodeling in pancreatic cancer. These structural features may contribute to the metabolic phenotype characteristic of PDAC, which can be captured in EUS-FNA–derived samples.

### 3.6 Differential lipid metabolites

**FIGURE 6:**
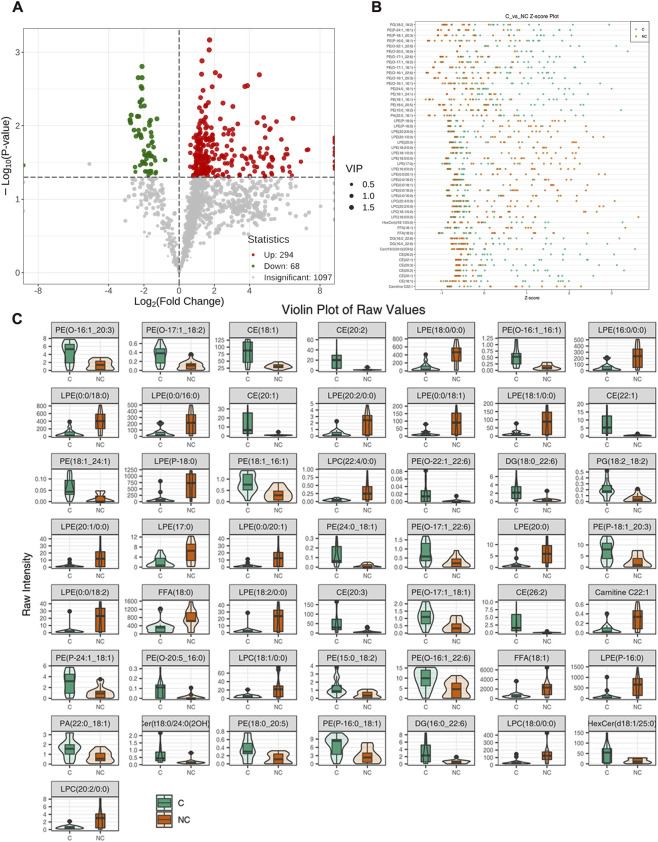
Identification and visualization of differential lipid species. **(A)** Volcano plot displaying significantly upregulated (red) and downregulated (green) lipids in tumor samples compared to controls. **(B)** Z-score heatmap illustrating abundance patterns of the top differential lipids between groups. **(C)** Violin plots showing the distribution of raw intensities for representative differential lipid species.

A total of 362 lipid species were identified as significantly altered between tumor and non-tumor tissues based on variable importance in projection (VIP >1), fold change >2 or <0.5, and adjusted p < 0.05 (Figure 6A). Among them, 294 lipids were upregulated and 68 were downregulated in tumor samples. Z-score visualization revealed distinct abundance patterns of these differential lipids across groups (Figure 6B). Representative violin plots demonstrated consistent upregulation of specific triglycerides and phospholipids in tumor tissues, highlighting their potential relevance in pancreatic cancer lipid remodeling (Figure 6C). Notably, differential lipids were predominantly upregulated in tumor tissues, with enrichment in triglycerides and membrane-associated phospholipids, suggesting a coordinated shift toward lipid accumulation and structural remodeling rather than isolated metabolic fluctuations, as observed in the present FNA-derived dataset.

### 3.7 Pathway enrichment analysis

**FIGURE 7:**
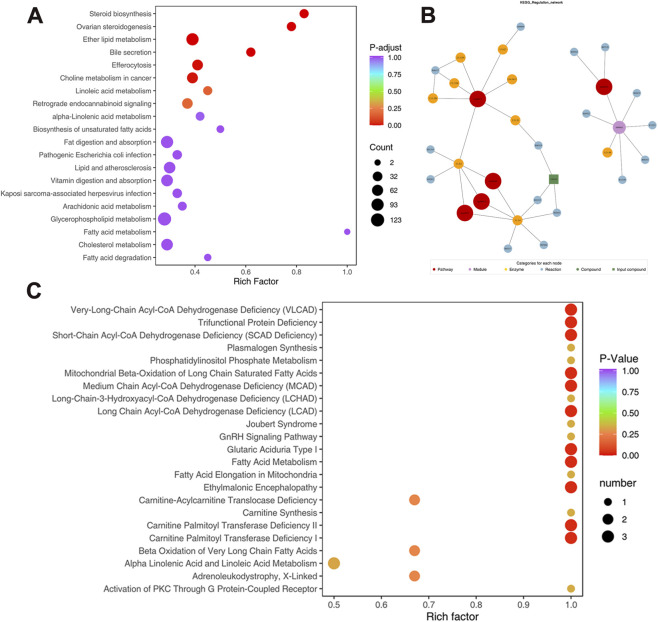
Pathway enrichment and network analysis of differential lipids. **(A)** KEGG pathway enrichment analysis of differential lipid metabolites, with bubble size representing metabolite count and color indicating adjusted P-values. **(B)** KEGG network visualization showing relationships between significant pathways, modules, and compounds. **(C)** HMDB enrichment analysis of differential lipid species, highlighting enrichment in specific metabolic contexts.

KEGG enrichment analysis identified multiple metabolic pathways significantly associated with the differential lipids, including steroid biosynthesis, glycerophospholipid metabolism, and linoleic acid metabolism (Figure 7A). KEGG network visualization revealed interconnected modules linking altered lipid metabolites to specific biochemical processes (Figure 7B). HMDB enrichment further confirmed that many differential lipids were involved in fatty acid oxidation and mitochondrial lipid metabolism pathways (Figure 7C). Collectively, enriched pathways converged on lipid synthesis, membrane remodeling, and fatty acid utilization, reinforcing the coordinated nature of metabolic reprogramming in PDAC.

### 3.8 Exploratory projection of the FNA-derived lipid signature to serum

**FIGURE 8:**
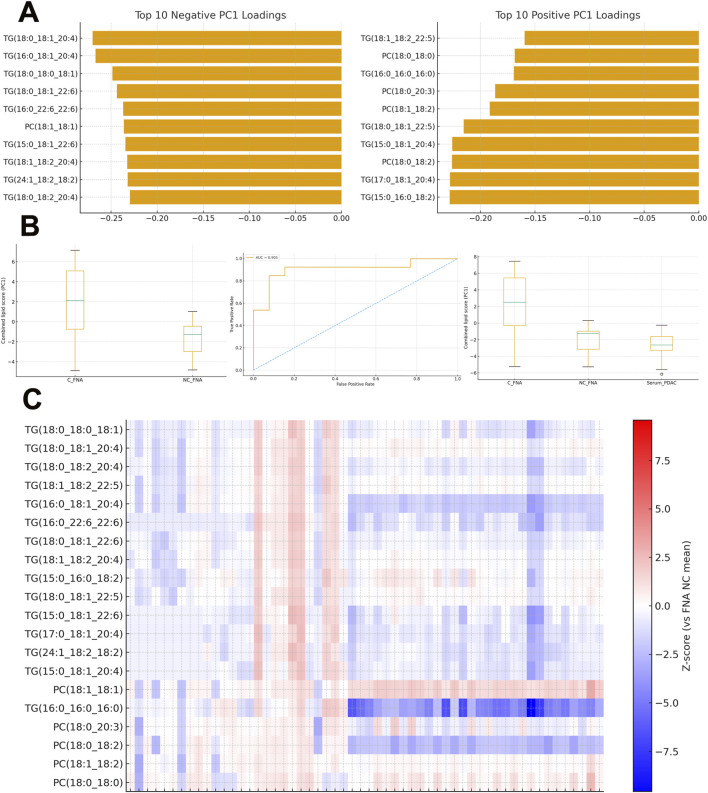
Exploratory evaluation of the FNA-derived lipid signature and its projection to serum samples. **(A)** Top positive and negative PC1 loadings derived from principal component analysis of differential lipids in FNA samples, representing the major contributors to tumor–non-tumor separation. **(B)** Distribution of the combined lipid score, constructed from 20 FNA-derived signature lipids, in tumor and adjacent non-tumor FNA samples. Receiver operating characteristic (ROC) analysis of the lipid score is shown. **(C)** Distribution of the same lipid score in serum samples, along with heatmap visualization of the 20 FNA-derived signature lipids projected onto serum samples.

To explore whether the FNA-derived lipid signature reflects broader metabolic alterations, we derived a composite lipid score based on 20 signature lipids identified from FNA specimens (Figure 8A). When applied to PDAC serum samples, the combined lipid score demonstrated a directional deviation relative to the reference range defined by adjacent non-tumor FNA tissues (Figure 8B).

Consistently, heatmap visualization of the 20 signature lipids revealed coordinated shifts in serum lipid profiles compared with the NC-defined reference pattern (Figure 8C). Although this analysis was exploratory and not intended for formal diagnostic validation, the observed trends suggest that elements of the FNA-derived lipid signature may extend beyond local tumor tissue and align with systemic metabolic alterations associated with PDAC.

## 4 Discussion

In this study, EUS-FNA–derived micro-biopsy samples can capture tumor-associated lipidomic remodeling in a preoperative setting. Despite the limited tissue quantity inherent to FNA sampling, LC–MS–based lipidomic profiling successfully captured coordinated alterations in tumor-associated lipid classes and structural features (Cajka and Fiehn, 2014). These findings suggest that lipid metabolic reprogramming in PDAC can be preserved at the micro-biopsy level and is suitable for high-sensitivity mass spectrometry analysis.

Lipid metabolic reprogramming is increasingly recognized as a fundamental feature of cancer cells, enabling them to support rapid proliferation, maintain energy homeostasis, and adapt to metabolic stress (Bian et al., 2021), and our findings suggest that these features can also be detected in EUS-FNA–derived micro-biopsy samples.

Most previous lipidomic studies in pancreatic cancer have used surgically resected tissues or serum samples (Wolrab et al., 2022). These materials differ from routine preoperative biopsy specimens. In our study, triglyceride accumulation and increased lipid unsaturation were also observed in EUS-FNA samples, indicating that these metabolic changes are present at the time of diagnosis and are likely intrinsic to tumor biology. This may reflect adaptive metabolic reprogramming, in which triglyceride accumulation serves as an energy reservoir and may also contribute to cellular adaptation under metabolic stress conditions (Bian et al., 2021). These features may also be preserved in micro-biopsy specimens obtained via FNA.

Notably, lipid alterations observed in tumor FNA samples were not randomly distributed across classes but were enriched in storage lipids and membrane-associated phospholipids, accompanied by elongation of fatty acyl chains and increased degrees of unsaturation, suggesting coordinated lipid metabolic remodeling in tumor tissues. These coordinated structural changes may reflect adaptive remodeling to support membrane biosynthesis, signal transduction, and proliferative demands in malignant cells as increased fatty acyl chain unsaturation is known to enhance membrane fluidity and facilitate dynamic cellular processes under stress conditions. Such architectural remodeling of lipid species has been implicated in oncogenic metabolic adaptation across multiple tumor types and may represent a common metabolic feature of malignant transformation (Rohrig and Schulze, 2016; Beloribi-Djefaflia et al., 2016; Tan et al., 2022), which can be captured using FNA-derived samples in a preoperative setting.

Although the present study was not designed for formal diagnostic validation, exploratory projection of the FNA-derived lipid signature to serum samples revealed directionally consistent alterations. This observation suggests that elements of tumor-associated lipid remodeling may extend beyond local tissue and reflect systemic metabolic perturbations in PDAC (Qin et al., 2020). Larger case–control cohorts will be required to determine the diagnostic and prognostic relevance of these findings.

Several limitations should be acknowledged. First, the sample size of the EUS-FNA cohort was modest and derived from a single center, which may limit generalizability. This limitation primarily reflects the inherent constraints of EUS-FNA sampling in clinical practice, where the amount of tissue obtained is limited and a portion of the sample is required for cytopathological examination, thereby restricting its availability for downstream lipidomic analysis. In addition, no independent validation cohort was included, and stage-specific or outcome-based analyses were not performed. Furthermore, while pseudo-targeted lipidomic profiling provided high analytical sensitivity, functional validation of specific lipid species was beyond the scope of the present study.

Despite these limitations, our findings suggest that EUS-FNA-derived micro-biopsy samples can capture biologically meaningful lipidomic signals, supporting the feasibility of applying lipidomics to minimally invasive diagnostic materials. Future investigations incorporating multi-center cohorts, longitudinal sampling, and mechanistic experiments will be necessary to further clarify the translational implications of lipid remodeling in PDAC.

In summary, this study demonstrates that EUS-FNA–derived micro-biopsy specimens can serve as a feasible platform for capturing tumor-associated lipidomic features in PDAC, supporting the feasibility of lipidomic profiling in a preoperative diagnostic setting. These findings establish a foundation for future translational studies exploring metabolic biomarkers in minimally invasive pancreatic cancer diagnostics.

## 5 Conclusion

This study suggests that EUS-FNA–derived micro-biopsy specimens can reliably capture lipidomic remodeling in PDAC in a preoperative setting. Tumor samples exhibited coordinated alterations in storage lipids and structural lipid features, including fatty acyl chain elongation and increased unsaturation. These findings support the feasibility of lipid profiling in routine diagnostic material and provide a foundation for future translational investigations in minimally invasive pancreatic cancer diagnostics.

## Data Availability

The original contributions presented in the study are included in the article/Supplementary Material, further inquiries can be directed to the corresponding authors.

## References

[B1] Antonova L. Paramanthan P. Falls T. Wedge M. E. Mayer J. Sekhon H. S. (2024). Molecular characterization and xenotransplantation of pancreatic cancer using endoscopic ultrasound-guided fine needle aspiration (EUS-FNA). Cancers (Basel) 16 (15), 2721. 10.3390/cancers16152721 39123450 PMC11311391

[B2] Ashida R. Kitano M. (2022). Endoscopic ultrasound-guided tissue acquisition for pancreatic ductal adenocarcinoma in the era of precision medicine. Dig. Endosc. 34 (7), 1329–1339. 10.1111/den.14344 35488448

[B3] Beloribi-Djefaflia S. Vasseur S. Guillaumond F. (2016). Lipid metabolic reprogramming in cancer cells. Oncogenesis 5 (1), e189. 10.1038/oncsis.2015.49 26807644 PMC4728678

[B4] Bian X. Liu R. Meng Y. Xing D. Xu D. Lu Z. (2021). Lipid metabolism and cancer. J. Exp. Med. 218 (1), e20201606. 10.1084/jem.20201606 33601415 PMC7754673

[B5] Biancur D. E. Paulo J. A. Małachowska B. Quiles Del Rey M. Sousa C. M. Wang X. (2017). Compensatory metabolic networks in pancreatic cancers upon perturbation of glutamine metabolism. Nat. Commun. 8, 15965. 10.1038/ncomms15965 28671190 PMC5500878

[B6] Cajka T. Fiehn O. (2014). Comprehensive analysis of lipids in biological systems by liquid chromatography-mass spectrometry. Trends Anal. Chem. 61, 192–206. 10.1016/j.trac.2014.04.017 25309011 PMC4187118

[B7] Cho S. Sung H. K. Nguyen K. Lei Y. Wannaiampikul S. Lee B. (2026). Lipidomic analysis of plasma extracellular vesicles from adiponectin deficient mice or metabolic syndrome patients reveals pro-oxidative and pro-inflammatory lipid signatures correlating with metabolic dysfunction. J. Extracell. Vesicles 15 (2), e70229. 10.1002/jev2.70229 41656969 PMC12884008

[B8] Du P. Wang Q. He Y. Yu H. Lin L. Zhang Z. (2024). Lipidomic profiling and storage-induced changes in cassava flour using LC-MS/MS. Foods 13 (19), 3039. 10.3390/foods13193039 39410074 PMC11475662

[B9] Halbrook C. J. Lyssiotis C. A. (2017). Employing metabolism to improve the diagnosis and treatment of pancreatic cancer. Cancer Cell 31 (1), 5–19. 10.1016/j.ccell.2016.12.006 28073003

[B10] Hewitt M. J. McPhail M. J. W. Possamai L. Dhar A. Vlavianos P. Monahan K. J. (2012). EUS-guided FNA for diagnosis of solid pancreatic neoplasms: a meta-analysis. Gastrointest. Endosc. 75 (2), 319–331. 10.1016/j.gie.2011.08.049 22248600

[B11] Horejsi K. Jin C. Vaňková Z. Jirásko R. Strouhal O. Melichar B. (2023). Comprehensive characterization of complex glycosphingolipids in human pancreatic cancer tissues. J. Biol. Chem. 299 (3), 102923. 10.1016/j.jbc.2023.102923 36681125 PMC9976472

[B12] Iwashita T. Uemura S. Ryuichi T. Senju A. Iwata S. Ohashi Y. (2024). Advances and efficacy in specimen handling for endoscopic ultrasound-guided fine needle aspiration and biopsy: a comprehensive review. Den. Open 4 (1), e350. 10.1002/deo2.350 38495467 PMC10941515

[B13] Kamphorst J. J. Nofal M. Commisso C. Hackett S. R. Lu W. Grabocka E. (2015). Human pancreatic cancer tumors are nutrient poor and tumor cells actively scavenge extracellular protein. Cancer Res. 75 (3), 544–553. 10.1158/0008-5472.CAN-14-2211 25644265 PMC4316379

[B14] Maher M. H. Treekitkarnmongkol W. Ghatak S. Dai J. Liu S. Nguyen T. (2025). An integrated multi-omics biomarker approach using molecular profiling and microRNAs for evaluation of pancreatic cyst fluid. Cancer Cytopathol. 133 (4), e70008. 10.1002/cncy.70008 40106268 PMC13493043

[B15] Manthopoulou E. Ramai D. Ioannou A. Gkolfakis P. Papanikolaou I. S. Mangiavillano B. (2023). Endoscopic ultrasound-guided tissue acquisition beyond the pancreas. Ann. Gastroenterol. 36 (3), 257–266. 10.20524/aog.2023.0797 37144012 PMC10152811

[B16] Naudin S. Sampson J. N. Moore S. C. Albanes D. Freedman N. D. Weinstein S. J. (2023). Lipidomics and pancreatic cancer risk in two prospective studies. Eur. J. Epidemiol. 38 (7), 783–793. 10.1007/s10654-023-01014-3 37169992 PMC11152614

[B17] Perazzoli G. García-Valdeavero O. M. Peña M. Prados J. Melguizo C. Jiménez-Luna C. (2023). Evaluating metabolite-based biomarkers for early diagnosis of pancreatic cancer: a systematic review. Metabolites 13 (7), 872. 10.3390/metabo13070872 37512579 PMC10384620

[B18] Qin C. Yang G. Yang J. Ren B. Wang H. Chen G. (2020). Metabolism of pancreatic cancer: paving the way to better anticancer strategies. Mol. Cancer 19 (1), 50. 10.1186/s12943-020-01169-7 32122374 PMC7053123

[B19] Rohrig F. Schulze A. (2016). The multifaceted roles of fatty acid synthesis in cancer. Nat. Rev. Cancer 16 (11), 732–749. 10.1038/nrc.2016.89 27658529

[B20] Shevchenko A. Simons K. (2010). Lipidomics: coming to grips with lipid diversity. Nat. Rev. Mol. Cell Biol. 11 (8), 593–598. 10.1038/nrm2934 20606693

[B21] Siegel R. L. Giaquinto A. N. Jemal A. (2024). Cancer statistics, 2024. CA Cancer J. Clin. 74 (1), 12–49. 10.3322/caac.21820 38230766

[B22] Siegel R. L. Kratzer T. B. Giaquinto A. N. Sung H. Jemal A. (2025). Cancer statistics, 2025. CA Cancer J. Clin. 75 (1), 10–45. 10.3322/caac.21871 39817679 PMC11745215

[B23] Tan Y. Li J. Zhao G. Huang K. C. Cardenas H. Wang Y. (2022). Metabolic reprogramming from glycolysis to fatty acid uptake and beta-oxidation in platinum-resistant cancer cells. Nat. Commun. 13 (1), 4554. 10.1038/s41467-022-32101-w 35931676 PMC9356138

[B24] Tempero M. A. Malafa M. P. Al-Hawary M. Behrman S. W. Benson A. B. Cardin D. B. (2021). Pancreatic adenocarcinoma, version 2.2021, NCCN clinical practice guidelines in oncology. J. Natl. Compr. Canc Netw. 19 (4), 439–457. 10.6004/jnccn.2021.0017 33845462

[B25] Wang G. Yao H. Gong Y. Lu Z. Pang R. Li Y. (2021). Metabolic detection and systems analyses of pancreatic ductal adenocarcinoma through machine learning, lipidomics, and multi-omics. Sci. Adv. 7 (52), eabh2724. 10.1126/sciadv.abh2724 34936449 PMC8694594

[B26] Wenk M. R. (2005). The emerging field of lipidomics. Nat. Rev. Drug Discov. 4 (7), 594–610. 10.1038/nrd1776 16052242

[B27] Wolrab D. Jirásko R. Cífková E. Höring M. Mei D. Chocholoušková M. (2022). Lipidomic profiling of human serum enables detection of pancreatic cancer. Nat. Commun. 13 (1), 124. 10.1038/s41467-021-27765-9 35013261 PMC8748654

